# The Connection Between Canine Fossa Topography and Facial Morphology

**DOI:** 10.3390/dj13060229

**Published:** 2025-05-22

**Authors:** Carol Antonio Dandoczi, Mugurel Constantin Rusu, Răzvan Costin Tudose

**Affiliations:** 1 Division of Anatomy, Department 1, Faculty of Dentistry, “Carol Davila” University of Medicine and Pharmacy, 050474 Bucharest, Romania; carol-antonio.dandoczi@drd.umfcd.ro (C.A.D.); razvan-costin.tudose0721@stud.umfcd.ro (R.C.T.); 2 Research Department, “Dr. Carol Davila” Central Military Emergency Hospital, 010825 Bucharest, Romania

**Keywords:** canine fossa, facial index, cephalometry

## Abstract

**Objectives:** This study investigates the canine fossa (CF) topography relative to the maxillary sinus and nasal fossa and examines its association with facial types, focusing on side and gender. **Methods:** One hundred CBCT scans were analysed bilaterally at the first (PM1) and second (PM2) premolar levels. The CF was classified according to its anatomical relationship with adjacent structures: type 1, where the CF lies predominantly lateral to the maxillary sinus; type 2, where it is lateral to the nasal fossa; and type 3, where the CF is positioned lateral to the nasal fossa inferiorly and to the maxillary sinus superiorly. Facial measurements (height and width) were used to calculate the facial index, classifying individuals into five facial types: hypereuroprosopic, euryprosopic, mesoprosopic, leptoprosopic, and hyperleptoprosopic. A statistical analysis assessed the correlation between CF types and facial morphology. **Results:** Types 1 and 3 were the most prevalent CF types, while type 2 was observed only at the PM1 site. The PM2 level showed a predominance of type 1, indicating a consistent anatomical association of the CF with the maxillary sinus. In contrast, the PM1 level demonstrated a higher prevalence of type 3, reflecting the presence of the CF in the walls of both the nasal fossa and maxillary sinus. Leptoprosopic facial type was strongly associated with type 3, while no significant associations of CF types were found for other facial types. The sample lacked hypereuroprosopic individuals (facial index < 79.9), while hyperleptoprosopic faces (facial index > 95) were present in 23% of cases. **Conclusions:** The CF topography varies significantly by premolar site and is influenced by facial proportions. Type-3 CFs correlate strongly with elongated (leptoprosopic) facial configurations; broader facial types were underrepresented in this sample.

## 1. Introduction

The definition of the canine fossa (CF) has evolved to reflect its anatomical complexity and evolutionary significance. Traditionally described as an infraorbital depression covering much of the maxillary zygomatic process [1], it has also been characterised by various authors as a variable cavity located beneath the infraorbital foramen [2], and a vertical depression above the canine root [3] or laterally to the canine eminence [4]. A more contemporary view integrates these perspectives, defining the CF as a variably extensive cavity on the anterior maxilla below the infraorbital foramen and lateral to the canine eminence, which acknowledges its anatomical diversity [5].

Clinically, the CF plays a significant role in maxillofacial surgical procedures [6]. Positioned between the infraorbital foramen and the alveolar process of the maxilla, it extends from the zygomaticomaxillary suture to the piriform aperture [1]. Its depth and the degree of maxillary sinus pneumatisation significantly influence the complexity of approaches such as the Caldwell–Luc and Denker techniques [7,8,9]. A deep CF, often associated with a thin and narrow face, can pose additional surgical challenges due to the need for more extensive bony removal [1]. Nevertheless, it was demonstrated that a large inferior nasal meatus may reach above the alveolar bone in the region of the maxillary premolars [10].

The present study aimed to analyse the CF aspect on CBCT scans at the level of the upper premolars, demonstrating that it may border not only the maxillary sinus but also the nasal fossa (specifically, the inferior nasal meatus) or, in some instances, both. In addition, this study seeks to explore the relationship between the CF and the facial type through cephalometric analysis.

## 2. Materials and Methods

This retrospective study analysed 100 archived CBCT scans, 52 from male and 48 from female patients. The database was fully anonymised; thus, only gender information was available, with no age or ethnicity retained. The investigation complied with the Declaration of Helsinki and received institutional ethics approval (No. 737/01 November 2024, Affiliation 2).

Scans were eligible if they (1) showed a fully dentate maxillary first- and second-premolar region bilaterally; (2) captured the entire CF, maxillary sinus, and nasal cavity within the field of view; (3) displayed no CBCT evidence of sinus or nasal-fossa pathology; and (4) were free of motion or truncation artefacts. Scans were excluded when any premolar was missing, image quality was faulty or truncated, or inflammatory, cystic, surgical, or other sinus/nasal pathology was visible.

The scan also had to clearly show all standard craniometric landmarks within the field of view for facial-index evaluation. Any scan lacking complete landmark visibility was excluded from this part of the study.

**Figure 1:**
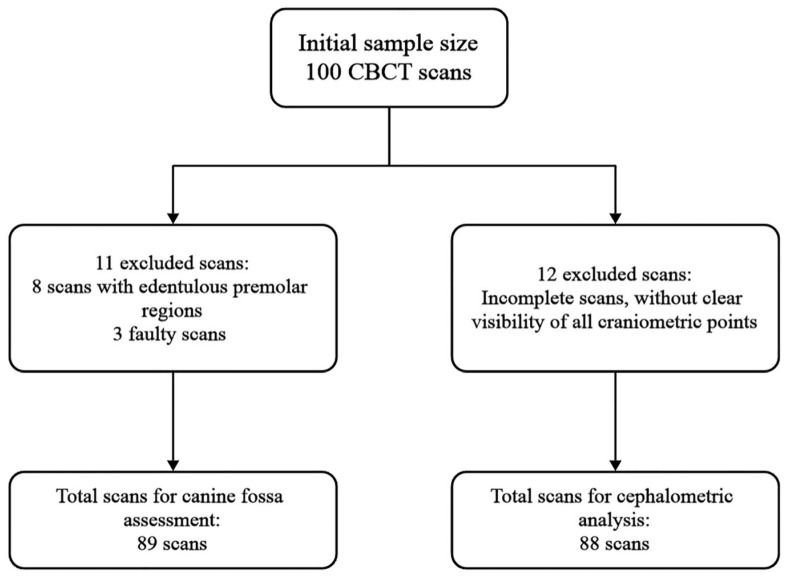
Flow diagram tracing the selection of scans from the initial to the final datasets used for canine fossa assessment and cephalometric analysis.

After applying these criteria, 89 scans remained for CF assessment; one lacked full cephalometric coverage, leaving 88 scans for the facial-index analysis (Figure 1).

All participants gave written consent to use their radiological data for research purposes. All scans were aligned to ensure accurate and reproducible measurements, with the skull oriented to achieve symmetry in the coronal view by visually aligning the frontozygomatic arches. The axial plane was aligned bilaterally, passing through the porion and orbitale craniometric points, corresponding to the Frankfurt horizontal plane. Images were acquired using a high-resolution iCat CBCT machine (Imaging Sciences International, Hatfield, PA, USA) with a 0.250 mm voxel resolution, a 130 mm field of view, and a 640 × 640 image matrix. The methodology has been detailed previously [11,12].

### 2.1. Canine Fossa Assessment

**Figure 2:**
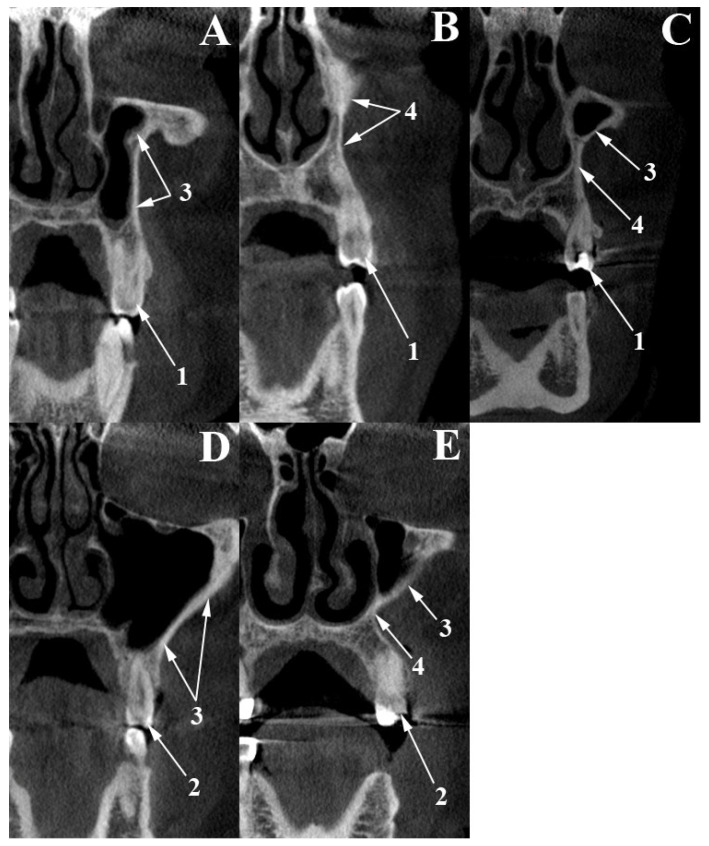
Left coronal views of the canine fossa (CF), through the first (**A**–**C**) and second (**D,E**) premolars, depicting the three types based on the CF’s medial relationship with either the maxillary sinus (MS) or the nasal fossa (NF). (**A**,**D**) CF type 1; (**B**) CF type 2; (**C**,**E**) CF type 3. 1. First premolar; 2. second premolar; 3. CF lateral to the MS; 4. CF lateral to the NF.

The Blue Sky Plan (version 4.12, Blue Sky Bio, LLC, Libertyville, IL, USA, 2021) software was used to evaluate the CF topography in the MPR panels. The assessment was performed bilaterally at the level of the 1st (PM1) and 2nd (PM2) premolars by two independent reviewers (MCR and CAD), who visually evaluated the relationship between the CF and adjacent anatomical structures. Cohen’s kappa was then calculated for each of the four measurements (right and left PM1 and PM2) to assess inter-rater reliability, followed by a pooled kappa derived from the combined contingency table to evaluate overall agreement across all sites. The qualitative scale for the interpretation of kappa coefficients has six levels, in which <0 is interpreted as no agreement, 0–0.20 as slight agreement, 0.21–0.40 as fair agreement, 0.41–0.60 as moderate agreement, 0.61–80 as substantial agreement, and 0.81–1.0 as almost perfect agreement [13]. The more experienced reviewer’s (MCR) assessment prevailed if discrepancies occurred. We excluded 11 cases with faulty scans or edentulous premolar regions, leaving 89 datasets for the final analysis. Based on its overall medial anatomical relationship, the CF region was classified into three types, assessed on coronal CBCT views, at the level of PM1 and PM2. After aligning each case, a coronal slice was positioned to intersect both the crown and root of the target premolar; the CF type was then recorded. In type 1 (antral type), the CF lies lateral to the maxillary sinus (Figure 2A,D). In type 2 (nasal type), the CF lies lateral to the nasal fossa’s dilated inferior nasal meatus (Figure 2B). In type 3 (antral–nasal type), the CF lies laterally both to the maxillary sinus superiorly and the dilated inferior nasal meatus inferiorly (Figure 2C,E). The classification was based on the appearance of the entire CF contour.

### 2.2. Cephalometric Measurements

**Figure 3:**
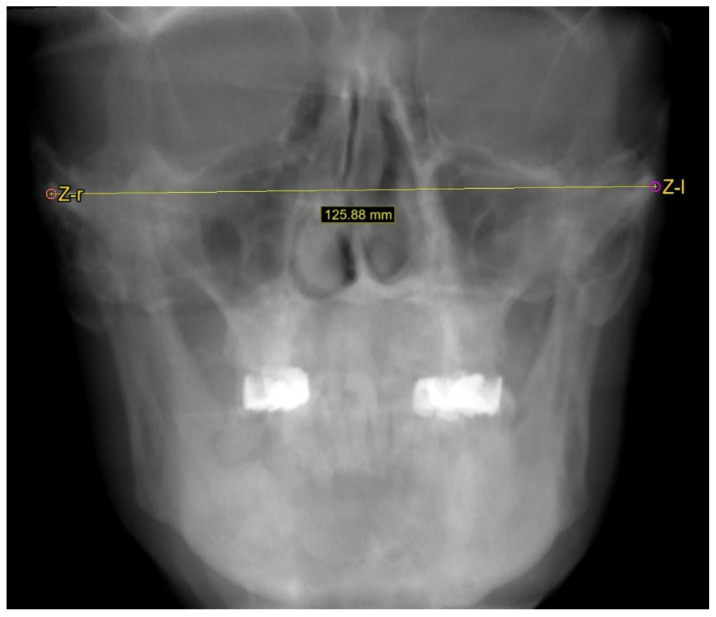
Frontal cephalometric radiograph. Z-r, Z-l—zygion points (right and left). The facial width was measured.

**Figure 4:**
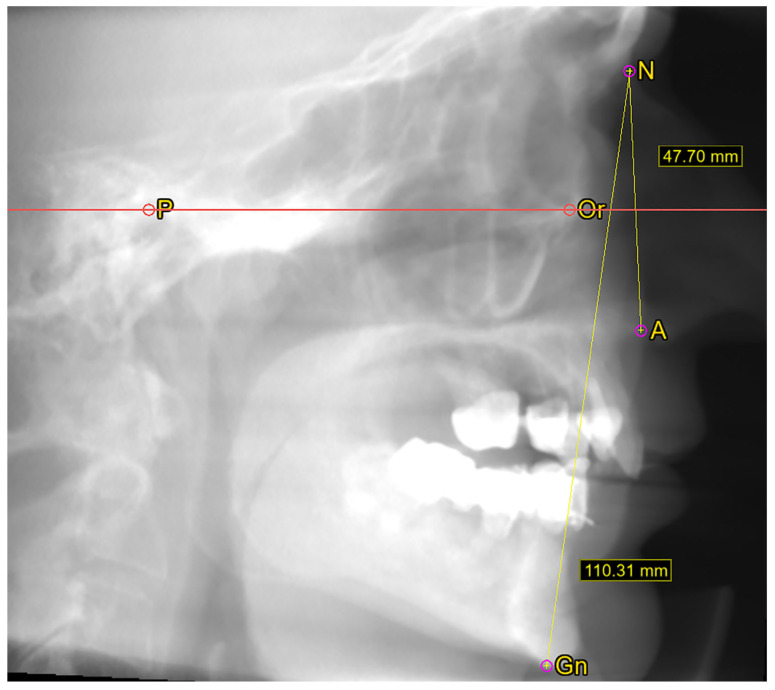
Lateral cephalometric radiograph. P—porion; Or—orbitale; N—nasion; ANS—anterior nasal spine; Gn—gnathion. The case was aligned with the Frankfurt plane positioned horizontally. Measurements were taken for both facial height and midface height.

Using the software’s cephalometric panel, specifically created for craniometric measurements, facial width was measured in the frontal incidence (zygion–zygion distance) (Figure 3), while facial height was measured in the sagittal incidence (nasion–gnathion) (Figure 4). Additionally, the midface height (nasion–subnasion) was also measured. The zygion point is the most lateral point of the facial contour on each zygomatic arch, identified through trial measurement [14]. Consequently, the facial width, or bizygomatic breadth, represents the most significant distance between the external aspects of the zygomatic arches [15]. The nasion is the craniometric point at the intersection of the internasal suture and the frontal bone, while the gnathion refers to the midpoint of the lower mandibular border [15]. The subnasion is defined as the point on the median line of the facial profile that represents the level of the nasal-fossa floor [16] and was marked at the tip of the anterior nasal spine. The facial index (FI) was calculated as FI = $\frac{Facial\;Height}{Facial\;Width}\times 100$. All measurements were in millimeters.

Face shapes were then classified into five types based on the FI, according to Bannister’s classification [17]. The face types were hypereuroprosopic (very broad face, FI ≤ 79.9), europrosopic (broad face, FI = 80.0–84.9), mesoprosopic (round face, FI = 85.0–89.9) leptoprosopic (long face, FI = 90.0–94.9), and hyperleptoprosopic (very long face, FI ≥ 95.0) [17].

Two reviewers (CAD and RCT) independently measured these distances to two decimal places. Twelve scans (11 males and 1 female) without clear visibility of all craniometric points were excluded from the analysis. The intraclass correlation coefficient (ICC) was then applied to assess inter-rater reliability using a two-way agreement-type model (ICC(A,1)) [18]. This approach was selected as we focused on absolute agreement, and each subject was measured once per rater. We interpreted the resulting ICC values based on the Koo and Li (2016) guidelines [19]: poor (0–0.5), moderate (0.5–0.75), good (0.75–0.9), and excellent (>0.9). Finally, the two sets of measurements were averaged to obtain a single dataset for further analysis.

### 2.3. Statistical Analysis

Our analysis included descriptive statistics performed in Microsoft Excel (version 2412) and detailed investigations using the RStudio (version 2024.04.1+748, RStudio Team, PBC, Boston, MA, USA, 2024) software. The analysis focused on cases eligible for both CF and facial type assessments (*n* = 79). Fisher’s exact test was used to assess the association between CF and facial type. Cases with bilaterally asymmetrical CF types were categorised as type A, which included 11 cases for PM1 and 10 cases for PM2. Symmetrical cases were assigned a single overall CF type. Since no cases had an FI below 80, the hypereuroprosopic type was excluded. The null hypothesis (H_0_) was that there is no association between CF type and facial type, while the alternative hypothesis (H_a_) proposed that such an association exists. A sensitivity analysis was performed by excluding asymmetrical cases to evaluate their impact on the results.

Separate evaluations were conducted for PM1 and PM2 sites. Since a statistically significant result was found only for the sensitivity analysis of the PM2 cases, we performed pairwise Fisher’s exact tests to compare the distribution of facial types between the two CF types (1 and 3) solely at this level. The null hypothesis (H_0_) for the pairwise tests was that the proportions of a specific facial type do not differ between CF types 1 and 3. In contrast, the alternative hypothesis (H_a_) proposed that the proportions vary significantly. Each pairwise test computed an odds ratio (OR) (indicating the likelihood of a facial type occurring in one group versus the other) and a two-sided *p*-value (assessing whether differences in proportions were statistically significant), with a significance threshold of 0.05. The presence of each specific facial type was treated as the “event”, while the absence of that facial type (the remaining facial types combined) was treated as the “non-event”. CF type 1 was used as the reference group for comparison against type 3. An OR greater than one indicated that the CF type was more likely to occur in PM2 type 3, while ORs less than one indicated a lower likelihood. Confidence intervals (CIs) for the ORs were calculated at the 95% confidence level. The ORs were derived from contingency tables using Fisher’s exact test, and the CIs were computed using a log-transformation method, which accounts for the sample distribution.

## 3. Results

### 3.1. Canine Fossa

The Cohen’s kappa test results indicated varying levels of agreement across different premolar sites. For the right PM1, the kappa value was 0.54, reflecting moderate agreement. The right PM2 showed a higher kappa value of 0.65, indicating substantial agreement. In contrast, the left PM1 had a kappa value of 0.38, signifying fair agreement, while the left PM2 had a kappa value of 0.55, indicating moderate agreement. The pooled Cohen’s kappa value across all sites was 0.58, demonstrating an overall moderate agreement between the two raters.

A total of 89 cases were analysed, comprising 46 males (51.7%) and 43 females (48.3%). At the right PM1 site, type 3 was the most frequent classification, observed in 45 cases (50.6%), followed by type 1 in 35 cases (39.3%), and type 2 in 9 cases (10.1%). For the right PM2 site, type 1 predominated with 69 cases (74.2%), while type 3 was observed in 20 cases (24.7%). Notably, type 2 was absent at this location. On the left side, the PM1 site similarly showed a higher frequency of type 3 (43 cases, 48.3%), followed by type 1 (33 cases, 37.1%) and type 2 (13 cases, 14.6%). The left PM2 was dominated by type 1 in 71 cases (79.8%), with type 3 identified in 18 cases (20.2%) and no instances of type 2.

Overall, types 1 and 3 were the most prevalent categories, varying by premolar site, while type 2 was the rarest configuration, observed only at the PM1 level. At the PM2 site, the absence of type 2 across all cases indicates that the nasal fossa was observed only in combination with the maxillary sinus. The predominance of type 1 at the PM2 level suggests that the CF primarily borders the maxillary sinus at this site. In contrast, the higher prevalence of type 3 at the PM1 site implies the simultaneous presence of the nasal fossa, particularly the inferior nasal meatus, and the maxillary sinus situated superiorly. No significant gender-based differences were observed in this analysis (Table 1).

In the bilateral asymmetry analysis, most cases were symmetrical, with 84.27% (75/89 cases) symmetry at the PM1 level and 88.76% (79/89 cases) at the PM2 level. At the PM1 level, 15.73% (14/89 cases) exhibited different bilateral types. Of these, 11.23% (10/89 cases) showed a combination of types 1 and 3, while 4.49% (4/89 cases) displayed a combination of types 2 and 3. Notably, no cases featured a type 1 and type 2 combination, indicating the absence of an antral-type CF on one side and a nasal-type configuration on the other. At the PM2 level, since type 2 was not observed in any case, the only bilateral combination was type 1 and type 3, found in 11.23% (10/89 cases).

### 3.2. Cephalometric Analysis

#### 3.2.1. Facial Width

The two reviewers showed an ICC(A,1) of 0.949, with a 95% CI of 0.923–0.966. This indicates a very high level of agreement for facial width measurements (*p* < 0.001).

In this dataset of 88 observations (41 males, 47 females), the mean value was 125.76, with a median of 125.69, indicating a near-symmetric distribution. The standard deviation was 6.69, reflecting moderate variability, with a range of 28.44 (minimum: 111.50, maximum: 139.94). No mode was identified, as no values were repeated. The dataset showed a slight positive skew (0.24), while the kurtosis value (−0.63) indicated a relatively flat distribution. The 95% CI for the mean was ±1.42, demonstrating a high precision level in the sample mean estimate.

Males had a larger mean facial width (130.02 mm) than females (122.04 mm), with slightly greater variability. Both distributions were slightly skewed toward lower values, but males showed a flatter distribution. The CI was narrower for females, reflecting a more precise estimate (Table 2).

#### 3.2.2. Midface Height

The ICC(A,1) was 0.893, suggesting high agreement between the two reviewers. The 95% CI of 0.831–0.932 and the significant *p*-value (*p* < 0.001) further support the reliability of these measurements.

The dataset, comprising 88 observations, had a mean of 52.07 and a median of 52.1, indicating an approximately symmetric distribution. The mode was 52.27, closely aligned with the mean and median. The standard deviation was 2.69, reflecting moderate variability, with a range of 15.73 (minimum: 46.08, maximum: 61.81). The skewness value of 0.48 indicated a slight positive skew, suggesting a mild right-tailed distribution, while the kurtosis value of 1.10 suggested slightly heavier tails and a sharper peak compared to a normal distribution. The 95% CI for the mean was ±0.57, indicating that the true population mean likely fell between 51.50 and 52.63.

The mean midface height was slightly more prominent in males (52.44 mm) than in females (51.74 mm). Males showed more significant variability, with a broader range (15.73 mm vs. 10.93 mm). Both distributions were slightly skewed, with males showing a more pronounced peak. The CIs indicated similar precision for both groups (Table 3).

#### 3.2.3. Facial Height

An ICC(A,1) of 0.914 (95% CI: 0.872–0.943) was observed, indicating substantial agreement between the reviewers. The result was highly significant (*p* < 0.001).

The dataset of 88 observations had a mean of 114.53 and a median of 115.11, indicating an approximately symmetric distribution, with no repeated values. The standard deviation was 5.05, with a range of 30.52 (minimum: 95.76, maximum: 126.27), reflecting moderate variability. The skewness of −0.56 indicated a longer left tail, meaning the distribution extended slightly toward lower values. The kurtosis of 1.56 suggested a sharper peak at the centre and slightly heavier tails, indicating the presence of some extreme values compared to a normal distribution. The 95% CI for the mean was ±1.07, indicating that the true mean likely fell between 113.46 and 115.60.

The mean facial height was higher in males (116.96 mm) than in females (112.42 mm). Males showed less variability, while females had a slightly wider range. The male distribution was slightly skewed toward higher values, while the female distribution was skewed toward lower values (Table 2).

#### 3.2.4. Facial Index

For the FI, the two reviewers achieved an ICC(A,1) of 0.904 (95% CI: 0.857–0.936). This also represents high inter-rater reliability (*p* < 0.001).

The dataset, consisting of 88 observations, had a mean of 91.24 and a median of 91.10, indicating a symmetric distribution with no repeated values. The standard deviation was 4.96, showing moderate variability, with a range of 24.13 (minimum: 80.30, maximum: 104.43). The skewness value of 0.29 indicated a slight positive skew, suggesting a small tendency toward higher values. The kurtosis value of −0.31 suggested a distribution that was slightly flatter than a normal distribution, with fewer extreme values. The 95% CI for the mean was ±1.05, indicating that the true mean likely fell between 90.19 and 92.29.

No individuals were classified as hypereuroprosopic (FI < 79.9), indicating the absence of very broad facial structures in the sample. The europrosopic category (FI: 80–84.9) included seven individuals (8%), representing moderately broad facial proportions. The mesoprosopic category (FI: 85–89.9) contained 29 individuals (33%), indicating that a significant portion of the sample had average facial proportions. The leptoprosopic category (FI: 90–94.9) included 32 individuals (36%), making it the most frequent classification and reflecting a predominance of relatively long and narrow faces. Lastly, 20 individuals (23%) were classified as hyperleptoprosopic (FI > 95), indicating the presence of very elongated facial structures in a notable portion of the sample. The mean FI was higher in females (92.21) compared to males (90.14), with both classified as leptoprosopic. Females had slightly lower variability (SD: 4.63) than males (SD: 5.15). The male distribution was moderately skewed toward higher values, while the female distribution was nearly symmetrical (Table 2).

### 3.3. Correlation Between the Canine Fossa and Facial Type

The results for PM1 showed no significant association between CF and facial type, regardless of whether asymmetrical cases (type A) were included or excluded. Fisher’s exact test yielded *p*-values of 0.8797 (without type A) and 0.8633 (with type A), both well above the significance threshold of 0.05, leading us to fail to reject the null hypothesis. In contrast, the PM2 analysis indicated a potential association when asymmetrical cases were excluded. With type A included, the *p*-value was 0.06523, slightly above the significance threshold, indicating no significant association. However, when the sensitivity analysis was conducted, the *p*-value dropped to 0.04338, below 0.05, leading to the rejection of the null hypothesis and suggesting a significant association between CF and facial type at the PM2 level. This indicates that asymmetrical cases may introduce variability that obscures the association. The absence of type 2 at PM2 may also simplify the analysis and strengthen the signal, although this limits the analysis to fewer categories than PM1.

To further investigate this association, pairwise Fisher’s exact tests were performed for PM2 (excluding type A). For europrosopic cases, the OR was 0.0 with a *p*-value of 1.0, indicating that type-3 CF has no association with europrosopic facial type. For mesoprosopic cases, the OR was 0.86 with a *p*-value of 1.0, showing no significant difference between CF types 1 and 3 for this facial type. A significant result was found for leptoprosopic cases (OR = 4.71, *p* = 0.022), indicating that a type-3 CF is 4.71 times more likely to be associated with leptoprosopic cases than a type-1 CF. Finally, for hyperleptoprosopic cases, the OR was 0.0, with a borderline *p*-value of 0.055. Despite no hyperleptoprosopic cases being associated with type 3, the relatively high occurrence in type 1 (16 cases) caused the distribution to appear imbalanced enough to approach statistical significance (Table 3).

These highlight that type 3 shows a significantly higher association with the leptoprosopic facial type, no meaningful differences for europrosopic and mesoprosopic, and a potential but inconclusive trend for hyperleptoprosopic. Together, the results suggest that PM2-level analyses are sensitive to including asymmetrical cases and may be influenced by the reduced complexity from the absence of type-2 CFs at the PM2 level.

## 4. Discussion

### 4.1. Canine Fossa Type

Most studies of the maxillary sinus have focused on the dimensions and volume of the maxillary sinus [20,21,22], addressing aspects such as the sinus floor [23], sinus septa [24], and its recesses, but have paid less attention to its anteromedial boundary and its relationship with the nasal cavity. Kian Ang et al. specify “The base of each maxillary sinus forms the inferior part of the lateral wall of the nasal cavity” [25], but do not determine where the transition occurs from the maxillary sinus to the bone and the nasal cavity. We have investigated the relationship between the CF, the maxillary sinus, and the nasal cavity to determine whether facial patterns influence this relationship. Our study reports the possibility of the nasal fossa reaching the CF at the PM1 and PM2 levels.

No studies correlate the CF with deeper structures, such as the nasal cavity or maxillary sinus, and facial parameters. There are studies examining the correlation between facial patterns and the volume of the maxillary sinus, a correlation accepted by many authors [26,27]. One study that analyses the dimensions of the nasal cavities, maxillary sinus, and facial skeleton emphasises these structures’ influence on each other [28].

In most cases, the anterior boundary of the maxillary sinus is most commonly observed in the PM1 area and with a higher frequency in the canine area compared to the PM2 area [29]. This observation indicates a tendency for anteromedial expansion, suggesting that the maxillary sinus is more often elongated rather than short. Our study also supports this finding, showing that the CF was most frequently located laterally to the maxillary sinus in types 1 and 3. Additionally, in the PM1 area, the maxillary sinus is narrower transversely, with a tendency to widen towards the distal region [30].

There are numerous measurements of the volume of the maxillary sinus. Sahlstrand-Johnson et al. estimate the volume using measurements in three planes and assess the wall thickness in the CF area [31]. The volume of the maxillary sinus varies by sex, but the thickness of this wall does not present significant differences between sexes [31]. According to Saquib-Abullais et al., facial patterns do not influence the thickness of the CF’s bony wall [32]. Their study identifies the most common facial pattern as leptoprosopic, similar to ours [32].

At the PM1 level, type-1 and type-3 CFs were common, while type 2 appeared only occasionally. At the PM2 level, type 2 was absent; type 1 predominated, and type 3 was less frequent than type 1. This distribution shows how the CF’s relationship to the nasal cavity and maxillary sinus shifts along the anteroposterior axis.

Mustian (1933) studied 100 human maxillary sinuses on dried skulls [33]. The author did not calculate prevalences for the observed anatomical landmarks [33]. He noted that a CF that appears to approach the lateral nasal wall can be misleading [33]. In such cases, the maxillary sinus either extends sagittally, with a strip below the CF, or ends abruptly immediately distal to the CF [33]. Therefore, to enter the maxillary sinus through the CF, it is preferable to approach the anterolateral aspect of the zygomatic process of the maxilla [33]. This opening should be high to avoid injury to the superior alveolar plexus and dental apices [33]. Topographically, this approach will be inferior and distal to the infraorbital foramen, which will avoid the branches of the infraorbital nerve and artery [33]. We agree with Mustian’s (1933) fairly reliable theorem that when the CF corresponds to the lateral nasal wall, the upper canines and premolars do not directly contact the maxillary sinus [33]. In such cases, a force misapplied against these teeth will push them into the CF or the inferior nasal meatus [33]. An attempt to open the maxillary sinus via the CF will create an opening of the inferior nasal meatus [29].

### 4.2. Cephalometric Analysis

The facial index obtained in our study is 91.24. Compared to other international studies (Table 4), Italy presents the highest facial index (97.9), which may indicate a favourable aesthetic or developmental standard compared to other populations [34]. On the other hand, Germany and Lithuania have lower scores, suggesting variations in the development and perception of facial indices in relation to culture and genetics [34]. Ogodescu et al.’s study, which indicated an average of 81.33 for a group aged 13–14 years, may highlight the evolution of facial indices during adolescence [35]. In Shetti et al.’s study, girls from Malaysia have a higher facial index (87.71) compared to boys (85.72), suggesting possible gender variations in the development of facial index [36]. In Indian studies, boys (87.19) exhibit a slightly higher facial index than girls (86.75) [36], which may reflect cultural or genetic trends. These observations suggest that a combination of sex, age, and cultural environment influences the facial index.

Our study found a significant distribution of facial types, with 32 (36%) mesoprosopic and 20 (23%) hyperleptoprosopic individuals, suggesting a trend toward balanced facial shapes. In comparison, the study by Nicoli et al. (2019) showed a predominance of the europrosopic type, with 532 (53.2%) europrosopic individuals, highlighting a notable difference in the use of a different scale for the facial index [38]. In a study by Kataria et al. (2013), nearly half of the subjects (46%) were leptoprosopic, which contrasts significantly with our results [37]. Additionally, a study by Pouya et al. (2021) highlighted a variation within the female group, where 44.35% were identified as mesoprosopic, suggesting a marked difference in facial morphology between sexes and populations [39]. These data underscore morphological variability and the necessity of considering different evaluation methods in comparative studies (Table 5).

### 4.3. Clinical Significance

This premolar area is often used in oral implantology for anchoring implants, frequently using specific angles to avoid the maxillary sinus [41,42], or for bicortical anchorage in the nasal wall [38]. In cases of marked bone atrophy, the available bone is insufficient, even in the CF area. Jensen et al. [43] provide an approach in which the implant anchorage is achieved in the nasal wall using a palatal technique. On the other hand, it has been emphasised that when the apical end of the implant reaches the inferior nasal meatus, both the nasal mucosa and the implant show no signs of inflammation [44].

Type 3, in which the CF is posterior and inferior to the maxillary sinus, was the most frequently observed in our study. This finding strongly correlates with the leptoprosopic facial pattern characterised by a narrow face. The likelihood of encountering the nasal fossa or maxillary sinus during bone addition techniques or implant placement significantly increases in such patients. Due to the lack of studies correlating all these aspects, we highlight the importance of performing a preoperative CBCT for analysing variant anatomy.

The clinical significance of this area is considerable, as it serves as the osteotomy site for accessing the maxillary sinus and for bone trephination in the bone addition technique known as sinus lift [45], as well as in ENT pathology [46,47]. Approaching the maxillary sinus can be achieved through various techniques, but the lateral approach remains the most commonly used [48,49,50]. In our study, type 3 was predominantly encountered at PM1, with 42 cases (42%) for females and 46 cases (46%) for males. This indicates a significant increase in the likelihood that the nasal meatus and maxillary sinus are located posteromedially to the CF. Conversely, for PM2, this trend is reversed, with type 1 being predominantly encountered.

The clinical importance is particularly significant in the surgical field, both in dentistry and in ENT specialties. This approach is utilised for accessing the maxillary sinus and involves an area with adequate bony structure for implant placement. Our study emphasises that the canine fossa, in addition to its proximity to the maxillary sinus, may also have a close spatial relationship with the inferior nasal meatus. This aspect could complicate access to the maxillary sinus as well as the placement of dental implants. Therefore, we recommend performing preoperative evaluations using CBCT to assess the canine fossa in three dimensions and in relation to adjacent areas.

### 4.4. Study Limitations

This study has a few limitations that should be acknowledged. First, demographic and clinical data beyond gender were unavailable due to the retrospective use of archived CBCT scans, preventing more detailed stratified analyses. Second, the absence of individuals classified as hypereuroprosopic (FI < 79.9) limits the ability to explore associations between broader facial morphologies and CF configurations comprehensively. Lastly, the study’s cross-sectional nature does not allow assessment of developmental changes across age groups.

## 5. Conclusions

This study emphasises the anatomical variability of the CF with the maxillary sinus and nasal fossa across different premolar sites. At the PM1 site, the CF most commonly bordered both the inferior nasal meatus and the maxillary sinus, indicating a complex, dual spatial relationship. In contrast, at the PM2 site, the CF primarily walled the maxillary sinus, with no cases involving the nasal fossa alone. The analysis of facial dimensions revealed a notable trend toward longer and narrower faces, as the leptoprosopic and hyperleptoprosopic facial types were the most prevalent in the sample. Additionally, elongated facial configurations were significantly associated with the CF extending toward the nasal fossa inferiorly while remaining superiorly adjacent to the maxillary sinus. These suggest that the CF’s topography is influenced by its anatomical positioning and overall facial configuration.

## Figures and Tables

**Table 1:** Side- and gender-specific prevalence of canine fossa topographic types at the first (PM1) and second (PM2) premolar sites. The 95% confidence intervals (CI) are shown.

Gender	Position	Side	Type	No. of Cases	Prevalence (%)	95% CI (%)
**Female**	PM1	Left	1	17	39.53	26.37–54.42
			2	6	13.95	6.56–27.26
			3	20	46.51	32.51–61.08
		Right	1	17	39.53	26.37–54.42
			2	4	9.30	3.68–21.60
			3	22	51.16	36.75–65.38
	PM2	Left	1	35	81.40	67.38–90.26
			2	0	0.00	0.00–8.20
			3	8	18.60	9.74–32.62
		Right	1	34	79.07	64.79–88.58
			2	0	0.00	0.00–8.20
			3	9	20.93	11.42–35.21
**Male**	PM1	Left	1	16	34.78	22.68–49.23
			2	7	15.21	7.57–28.22
			3	23	50.00	36.12–63.88
		Right	1	18	39.13	26.39–53.54
			2	5	10.87	4.73–23.04
			3	23	50.00	36.12–63.88
	PM2	Left	1	36	78.26	64.43–87.74
			2	0	0.00	0.00–7.71
			3	10	21.74	12.26–35.57
		Right	1	35	76.09	62.06–86.09
			2	0	0.00	0.00–7.71
			3	11	23.91	13.91–37.94

**Table 2:** Cephalometric analysis based on gender.

Gender	Male				Female			
Metric	Facial Width	Midface Height	Facial Height	Facial Index	Facial Width	Midface Height	Facial Height	Facial Index
Mean	130.02	52.44	116.96	90.14	122.04	51.74	112.42	92.21
Standard Error	0.95	0.44	0.61	0.80	0.69	0.37	0.73	0.68
Median	130.08	52.68	116.71	88.48	121.56	51.58	112.16	92.09
Standard Deviation	6.07	2.81	3.90	5.15	4.72	2.57	5.01	4.63
Sample Variance	36.83	7.87	15.20	26.48	22.26	6.59	25.15	21.47
Kurtosis	−1.12	2.37	0.74	−0.33	−0.74	−0.07	1.45	0.52
Skewness	−0.16	0.37	0.29	0.69	−0.13	0.57	−0.73	0.02
Range	20.38	15.73	18.06	21.19	19.66	10.93	25.63	24.13
Minimum	119.56	46.08	108.22	80.30	111.50	47.58	95.76	80.30
Maximum	139.94	61.81	126.27	101.49	131.15	58.51	121.38	104.43
Confidence Level (95%)	1.92	0.89	1.23	1.62	1.39	0.75	1.47	1.36

**Table 3:** Pairwise Fisher’s exact test for the second premolar site, after excluding asymmetrical cases. NA—not available.

Facial Type	Occurrences in Type-1 Canine Fossa	Occurrences in Type-3 Canine Fossa	Odds Ratio (OR)	95% Confidence Interval	*p*-Value
Europrosopic	3	0	0	NA	1
Mesoprosopic	21	4	0.86	0.31–4.35	1
Leptoprosopic	17	8	4.71	1.18–18.72	0.022
Hyperleptoprosopic	16	0	0	NA	0.055

**Table 4:** Studies reporting facial indices in different populations. SD: standard deviation.

	Population Sample	Mean (SD)	Observations
Our study	Romania	91.24 (4.96)	-
Ritz-Timme et al. [34]	Germany	86.4 (5.2)	-
	Italy	97.9 (7.7)	-
	Lithuania	80.4 (5.0)	-
Kataria et al. [37]	Sindhi	92.89	-
Ogodescu et al. [35]	Romania	81.33 (3.55)	Age group 13–14.5 years
Shetti et al. [36]	Malaysian	85.72 (5.4)	Male group
	Malaysian	87.71 (5.1)	Female group
	Indian	87.19 (5.2)	Male group
	Indian	86.75 (6.3)	Female group

**Table 5:** Studies reporting facial types in different populations, based on gender.

	Hypereuroprosopic	Europrosopic	Mesoprosopic	Leptoprosopic	Hyperleptoprosopic	Observations
Our study	0	7 (8%)	29 (33%)	32 (36%)	20 (23%)	-
Nicoli et al. (2019) [38]	190 (19%)	532 (53.2%)	216 (21.6%)	56 (5.6%)	6 (0.6%)	Using a different scale of facial index
Kataria et al. (2013) [37]	0	2 (2%)	7 (7%)	46 (46%)	45 (45%)	Study conducted on male group
Ozsahin et al. (2016) [40]	85 (18.1%)	166 (35.3%)	156 (33.2%)	41 (8.7%)	22 (4.7%)	Male group
	83 (15.6%)	183 (34.33%)	183 (34.33%)	63 (11.8%)	21 (3.94%)	Female group
Pouya et al. (2021) [39]	7.1%	21.8%	36%	25.3%	9.8%	Male group
	4.46%	34.23%	44.35%	13.34%	3.62%	Female group

## Data Availability

The datasets used and analysed during the current study are available from the corresponding author upon reasonable request.
